# Construction of a taste-blind medaka fish and quantitative assay of its preference–aversion behavior

**DOI:** 10.1111/j.1601-183X.2008.00433.x

**Published:** 2008-11

**Authors:** Y Aihara, A Yasuoka, S Iwamoto, Y Yoshida, T Misaka, K Abe

**Affiliations:** † Department of Applied Biological Chemistry, Graduate School of Agricultural and Life Sciences, The University of TokyoTokyo; ‡ Department of Biological Engineering, Maebashi Institute of TechnologyMaebashi; § Faculty of Applied Biological Sciences, Gifu UniversityGifu, Japan

**Keywords:** Feeding behavior, G-protein, phospholipase C-beta 2, signal transduction, taste receptor, transgenic fish

## Abstract

In vertebrates, the taste system provides information used in the regulation of food ingestion. In mammals, each cell group within the taste buds expresses either the T1R or the T2R taste receptor for preference–aversion discrimination. However, no such information is available regarding fish. We developed a novel system for quantitatively assaying taste preference–aversion in medaka fish. In this study, we prepared fluorescently labeled foods with fine cavities designed to retain tastants until they were bitten by the fish. The subjects were fed food containing a mixture of amino acids and inosine monophosphate (AN food), denatonium benzoate (DN food) or no tastant (NT food), and the amounts of ingested food were measured by fluorescence microscopy. Statistical analysis of the fluorescence intensities yielded quantitative measurements of AN food preference and DN food aversion. We then generated a transgenic fish expressing dominant-negative Gα_i2_ both in T1R-expressing and in T2R-expressing cells. The feeding assay revealed that the transgenic fish was unable to show a preference for AN food and an aversion to DN food. The assay system was useful for evaluating taste-blind behaviors, and the results indicate that the two taste signaling pathways conveying preferable and aversive taste information are conserved in fish as well as in mammals.

On capturing food in their mouth, animals immediately need to identify whether it contains nutritious or toxic compounds. However, any tastant, nutritious or toxic, is received by taste buds located at the peripheral end of the taste system (Kinnamon *et al.* 1985; Northcutt 2004). Information relating to taste stimuli is transmitted to taste nerves and then to the central nervous system. The behavioral response to this taste signal transmission is either ingestion or ejection.

Recent studies have uncovered the molecular mechanisms underlying the initial step in taste reception. Mammalian T1Rs and T2Rs, which are G-protein coupled receptors (GPCRs), are expressed in taste bud cells (Adler *et al.* 2000; Hoon *et al.* 1999). T1R heteromers are activated by sugars and amino acids, which are preferable tastants, while T2Rs are activated by alkaloids that elicit aversive behavior (Chandrashekar *et al.* 2000; Nelson *et al.* 2001, 2002). T1Rs and T2Rs, when activated, transduce taste signals to G-proteins (Kusakabe *et al.* 2000; Wong *et al.* 1996) and then to the effector enzyme, namely phospholipase C-β 2 (PLC-β 2) (Adler *et al.* 2000). Because PLC-β2 knockout mice have reduced sensitivity to both T1R and T2R ligands (Zhang *et al.* 2003), this enzyme may be located at a critical point in the signaling pathways for the transduction of preferable and aversive tastes. Interestingly, these taste signaling pathways seem to be conserved in a wide variety of vertebrates ranging from mammals to fish (Ishimaru *et al.* 2005; Yasuoka *et al.* 2004).

Small fish species such as zebrafish (*Danio rerio*) and medaka fish (*Oryzias latipes*) are recognized as useful model animals for studying the involvement of the nervous system in sensory perception as their nervous systems consist of a small number of cells (Chapouton & Bahy-Cuif 2004; Schier *et al.* 1996). In addition, the small genome sizes and low redundancy of gene function are advantages when investigating sensory signaling at the molecular level (Crollius & Weissenbach 2005; Furutani-Seiki *et al.* 2004). Fish perceive amino acids, nucleotide-related substances and carbonic acids as taste stimulants (Kasumyan & Doving 2003; Kohbara *et al.* 1992; Marui & Caprio 1992; Valentincic & Caprio 1994; Yoshii *et al.* 1979). Their taste nerves also respond to quinine hydrochloride, caffeine and denatonium benzoate, which are perceived as bitter by humans (Chandrashekar *et al.* 2000; Ogawa *et al.* 1997). We have recently reported that fish T1Rs and T2Rs are activated by amino acids and denatonium, respectively (Oike *et al.* 2007), suggesting that fish and mammals share a common mechanism for taste discrimination. The fish is a useful model organism for studying the vertebrate taste system. In particular, the development of a quantitative assay system for evaluating taste preference–aversion behavior in small fish species is required. Here, we report the development of such a system that is applicable to medaka fish, and the construction of a transgenic, taste-blind phenotype in the species for confirming the utility of this system.

## Materials and methods

### Fluorescent dye-labeled food

The preferred tastant solution (AN solution) contained 100 mm glycine, 100 mml-serine, 100 mml-proline, 100 mm monosodium l-glutamate and 20 mm inosine monophosphate (IMP) disodium salt. The aversive tastant solution was composed of 100 mm denatonium benzoate (DN solution). An aqueous phase solution was prepared by dispersing 15 ml of tastant solution or water (for NT food) in 300 ml of 2% sorbitan monooleate (Emasol O-120V; Kao, Tokyo, Japan) at 60°C. Approximately 30 g of tripalmitin was melted at 70°C and mixed with 1.5 ml of 10 mm DiIC_12_(3) (Molecular probes D-383; Invitrogen, Carlsbad, CA, USA) ethanol solution to form a uniform molten mass (lipid phase). This was then poured into the aqueous phase at 60°C and stirred for 5 min at 10 000 r.p.m., using vacuum emulsification equipment (PVQ-3UN; Mizuho Industrial, Osaka, Japan). To prevent the development of coalesced droplets in the emulsion, 150 g of potato starch was added and the mixture was stirred quickly to encourage gelatinization. The mixture was then steamed for 5 min to allow the starch to solidify, and the sample was cooled rapidly in liquid nitrogen and then dried under a vacuum. The dried sample was milled and particles with a small diameter of 150–212 μm were selected. This size is slightly smaller than the mouth size of juvenile fish. To prepare diluted DN food, NT food was spread on a stainless steel sieve, dipped in denatonium solution or water (for NT^#^ food) and immediately dried. The dried mass was milled into particles with a diameter of 150–212 μm.

### Feeding assay

**Figure 1:**
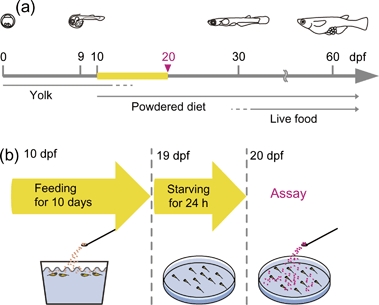
An experimental design for evaluating the feeding behavior of medaka fish (a) As they grow, medaka fish change their feeding behavior. After hatching at around 9 days postfertilization (dpf), the larvae begin to ingest a powdered diet as well as nutrients in the yolk sac, which is depleted at around 12 dpf. After 30 dpf, juvenile fish begin to ingest both a powdered diet and a live food (*Artemia salina*). Under laboratory conditions, the fish mature at around 60 dpf. (b) Preparing medaka fish for the behavioral assay. After hatching, larvae were placed in the maintenance system and fed the powdered diet until 19 dpf. They were then isolated in a Petri dish, starved for 24 h and subjected to the behavioral assay using fluorescently labeled food.

Medaka fish (Cab strain) were maintained in a system that has been described in an earlier publication (Fig. 1a) (Aihara *et al.* 2007). Fertilized eggs were collected and incubated at 28°C for 9 days in water containing 0.03% synthetic sea salt (Rohtomarine; Rei-sea, Tokyo, Japan) and 1 p.p.m. methylene blue. At 10 days postfertilization (dpf), hatched larvae were transferred to a maintenance system and raised on a powdered diet (Ayu Supergold 0; Oriental Yeast, Tokyo, Japan). At 19 dpf, juveniles were isolated and starved in a 90-mm Petri dish for 24 h at 25 ± 1°C. Then, 10 fish in each group were transferred to a new dish containing 40 ml of 0.1× balanced salt solution (17.0 mm NaCl, 0.4 mm KCl, 0.2 mm CaCl_2_, 0.3 mm MgSO_4_, 0.24 mm NaHCO_3_, pH 7.5 ± 0.1) and fed approximately 2 mg of fluorescent dye-labeled food for 150 seconds at 24.5 ± 0.5°C (Fig. 1b). The fish were killed in iced water and stored at −20°C until the quantification of food intake.

### Quantification of ingested food

Fish were mounted in a 96-well plastic plate, and observed under a fluorescence microscope (IX70; Olympus, Tokyo, Japan) equipped with an ND12.5 filter, excitation filter (520–550 nm) and emission filter (580IF). Images of the fish were obtained using a DP70 digital camera system (Olympus, Tokyo, Japan) with a 0.1 seconds exposure time and ISO200 sensitivity. We determined the fluorescence intensity (FI) values by integrating pixel values from images of the abdominal region using Photoshop CS2 (Adobe Systems, San Jose, CA) and the Scion Image Beta 4.0.2 software (Scion, Frederick, MD, USA; http://www.scioncorp.com/). A standard curve generated from InSpeck™ orange fluorescent microspheres (Invitrogen) was used to process the data, which were then submitted for statistical analysis. To extract DiIC_12_(3) from the bodies of the fish, each was homogenized in 200 μl of extraction solution (75% ethanol and 1% acetic acid) and centrifuged. This procedure was repeated, the 200 μl of supernatant from each extraction was pooled and the resulting 400 μl solution was filtered using Ultrafree-MC HV (Millipore, Billerica, MA, USA). A 100-μl aliquot was subjected to reverse-phase high-performance liquid chromatography (HPLC) analysis using a Shodex Rspak RP18-415 column (4.6 × 150 mm, 6 μm; Showa Denko K. K., Tokyo, Japan). The sample was eluted with a solution containing 50% methanol and 1% acetic acid at a flow rate of 1 ml/min at ambient temperature. DiIC_12_(3) was detected using a fluorescence detector with an excitation wavelength of 550 nm and an emission wavelength of 604 nm. The fluorescence was quantified based on the integrated peak area of the chromatogram with a retention time of 8.4 min. To obtain standards, the same analyses were performed against a given amount of NT food.

### Food intakes of wild-type medaka fish

We performed the assay four times and selected fish for further analysis with eye diameters of 460–560 μm as this measurement was closely proportional to their body size. The sample size for each test was calculated based on a type I error rate of 5% and a type II error rate of 20%. The between-food differences in mean FI values were analyzed by Dunnett's multiple comparison test using KyPlot 3.0 (KyensLab, Tokyo, Japan).

### G_i2_S47C transgenic fish

**Figure 4:**
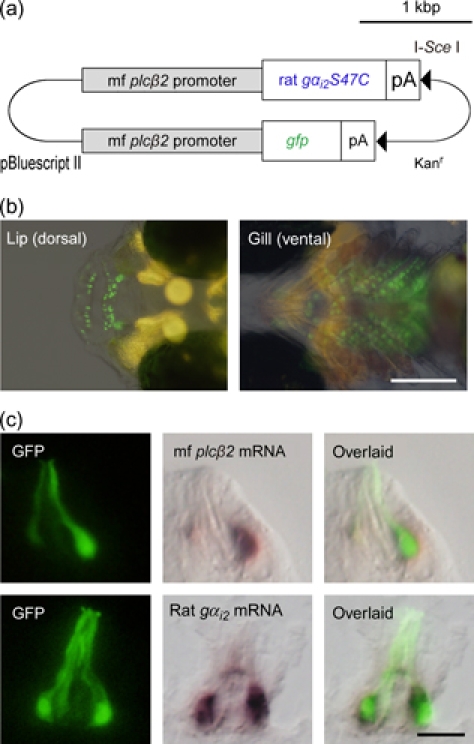
Generation of transgenic medaka fish expressing a dominant-negative form of the Gα_i2_ subunit in taste receptor cells (a) The construct contains two medaka *plc-β2* promoters that regulate rat *gα_i2_S47C* mutant and *gfp* genes. The two genes are in an inverted configuration with an *I-Sce*I meganuclease site at each 3′ end. Scale bar: 1 kbp. (b) The transgenic fish express GFP in the regions where taste buds are distributed. GFP was detected in the lip (left panel) and pharyngeal regions (right panel) of the transgenic fish (10 dpf). Images were obtained by overlaying fluorescence images on bright-field images. Scale bar: 200 μm. (c) Coexpression of the transgenes and endogenous *plc-β2* gene in the taste bud cells. In each experiment, fluorescence microscopy (panels in the left column) and *in situ* hybridization analyses (panels in the center column) were performed using the same section (upper or lower panels). The GFP signals were detected only in the cells expressing the endogenous *plc-β2* gene (upper panels). The cells expressing GFP also expressed rat *Gα_i2_* mRNA (lower panels). Scale bar: 10 μm.

Rat Gα_i2_ cDNA was obtained using the method described by Kusakabe *et al.* (2000). The cDNA fragment from the initiation codon to the Ser 47 codon was mutagenized using a *TaKaRa LA Taq* PCR kit (Takara Bio, Tokyo, Japan) and the primers 5′-gcgatatcatgggctgcaccgtgagcgccga-3′ and 5′-gcgatatcgTgcaccatcgtcaagcagatga-3′, producing an *Apa*LI recognition site. The cDNA fragment from the Ser 47 codon to the termination codon was also mutagenized using the same kit and the primers 5′-gcgatatctcagaagaggccacagtccttca-3′ and 5′-gcgatatcgtgcActtccctgattctccagc-3′, producing also an *Apa*LI recognition site. These fragments were digested by *Apa*LI and *Eco*RV and subcloned into the *Eco*RV site of pBluescript II SK(−) (Stratagene, La Jolla, Ca, USA) to obtain full-length rat Gα_i2_S47C cDNA. The cDNA was excised by *Eco*RV and inserted into the *Bam*HI site of p*mfplcb2*-1.6 kb (Aihara *et al.* 2007) to produce a *mfplcb2*-1.6 kb-rG_i2_S47C fragment. p*mfplcb2*-1.6 kb-EGFP<Kan> (Aihara *et al.* 2007) was used as a backbone vector. The SV40 polyadenylation signal for the rat Galpha_i2_S47C transcript was first subcloned into the *Sal*I site of p*mfplcb2*-1.6 kb-EGFP<Kan>. The *mfplcb2*-1.6 kb-rG_i2_S47C fragment was then excised by *Not*I and *Sma*I and inserted into the *Xho*I site of *mfplcb2*-1.6 kb-EGFP<Kan>, linking the SV40 polyadenylation signal to produce the final construct (Fig. 4a). Injection of the transgene and establishment of transgenic lines were performed using the method described previously (Aihara *et al.* 2007). Briefly, three G_0_ fish exhibiting the green fluorescent protein (GFP) signal were crossed with wild-type fish to obtain the F_1_ generation. Two lines were found to inherit the transgene, and the one exhibiting the most intense GFP signal was crossed with wild-type fish to produce the F_3_ and F_4_ generations. Mainly F_4_ fish were used in the food preference–aversion assay. The siblings exhibiting no GFP signal were used as wild-type controls.

### *In situ* hybridization

The digoxygenin-labeled antisense riboprobes were synthesized using the partial cDNA fragment encoding medaka PLC-β2 (Ile 369 to Ser 616, accession number AB254242) and full-length rat Gα_i2_S47C cDNA as templates. The head portion of each adult fish was removed and fixed in phosphate-buffered saline (PBS) containing 4% neutralized paraformaldehyde for 18 h at 4°C. They were then delipidated in 95% ethanol for 4 h at room temperature, decalcified in 0.5 m EDTA for 18 h at 4°C, embedded in Tissue–Tek O.C.T. compound (Sakura Finetechnical Co., Ltd., Tokyo, Japan) in vapors of liquid nitrogen and cryosectioned at a thickness of 4 μm. GFP fluorescence observation, followed by *in situ* hybridization, was performed as described by Aihara *et al.* (2007). The sections were incubated in a solution containing 100 mm Tris–HCl pH 7.5, 50 mm EDTA and 20 μg/ml proteinase K for 5 min. This was followed by treatment with PBS containing 2 mg/ml glycine, postfixation in PBS containing 4% neutralized paraformaldehyde for 10 min and acetylation with a solution containing 100 mm triethanolamine, 0.4% HCl and 0.25% acetic anhydrate for 10 min at room temperature. The sections were then hybridized with probes in a solution containing 50% formamide, 5× sodium chloride/sodium citrate (SSC), 5× Denhardt's solution, 500 μg/ml salmon testes DNA, 250 μg/ml torula tRNA and 1 mm dithiothreitol at 58°C for 36–40 h. After hybridization, the sections were washed twice in 5× SSC for 5 min at 58°C, and then washed twice in 0.2× SSC for 30 min at the same temperature. The probe was detected by incubating the sections in 0.5% blocking reagent (Roche Diagnostics, Indianapolis, IN, USA) in Tris-buffered saline containing a 1:500 dilution of anti-digoxigenin Fab fragments conjugated with alkaline phosphatase (Roche Diagnostics) for 1 h. This step was followed by a colorization reaction in a solution containing 100 mm Tris–HCl pH 9.5, 100 mm NaCl, 5 mm MgCl_2_, 0.2 mg/ml nitroblue tetrazolium and 0.05 mg/ml 5-bromo-4-chloro-3-indolyl phosphate, for 12 h at room temperature.

### Food intakes of G_i2_S47C transgenic fish

The preference assay was performed three times and the aversion assay twice using F_4_ transgenic fish and wild-type siblings. Statistical analysis was applied to selected fish with an average eye diameter of 500–510 μm. A two-way factorial analysis of variance (anova) with genotype and food as factors was used. To detect between-food differences, we used Aspin–Welch's *t*-test as a *post hoc* analysis. All data were analyzed using KyPlot 3.0 (KyensLab), with the significance level set at *P* < 0.05.

## Results

### Preparing fish for the feeding assay

Medaka fish change their feeding behavior during growth. After hatching, the larvae consume a powdered diet along with the nutrients in the yolk sac (Fig. 1a). The yolk sac disappears at around 12 dpf, and the larvae become increasingly more responsive to food. Supporting this, we detected more intense *plc-β2* expression in the taste bud cells at this stage than at the 9-dpf stage (unpublished data). The larvae also show food preferences. They examine an object on the surface of the water by biting into it, and, depending on their preference, either ingest or eject it. Fish are maintained on a powdered diet until 30 dpf and are then supplied with plankton as the main feed for the rest of their lives (Fig. 1a).

It seems that 20 dpf juvenile fish were suitable for use in our feeding assay as they were sufficiently developed to exhibit constant feeding behavior, yet possessed a transparent body that allowed fluorescence observations of the gastrointestinal tract. At 19 dpf, fish were isolated from the maintenance system, starved for 24 h in a dish, supplied with the fluorescently labeled food for 150 seconds in a new dish and subjected to the evaluation of food intake as follows (Fig. 1b).

### Designing fluorescently labeled food and its detection in the gastrointestinal tract of medaka fish

**Figure 2:**
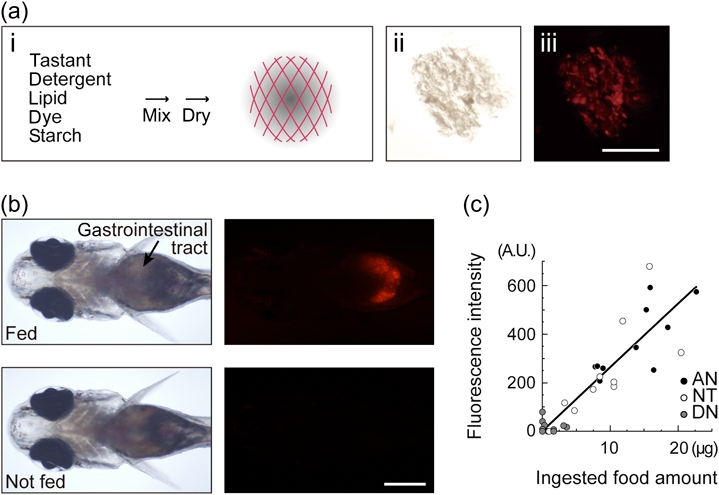
Designing fluorescent dye-labeled foods for medaka fish and detecting the food in the gastrointestinal tract (a) DiIC_12_(3)-labeled starch particles as labeled food: (i) schematic drawing, (ii) observation in the bright field and (iii) observation under fluorescence excitation. Each food particle forms a porous network in which starch, detergent, lipids, lipophilic dye and tastants are homogenously contained. Scale bar: 200 μm. (b) Images of a 20-dpf fish fed the fluorescent dye-labeled food (upper panels), and a fish that was not fed the food (lower panels). Bright-field images (panels in the left column) and fluorescence images (panels in the right column) are shown. The red fluorescence derived from DiIC_12_(3) was detected only in the abdominal region of the fish that were fed the food (upper panel). Scale bar: 500 μm. (c) Correlation between FI values and the amount of food ingested by the fish. The fish were fed foods containing no tastant (NT), amino acid–IMP mix (AN) and denatonium benzoate (DN). Each FI value is the sum of the pixel values in the fluorescence image of each fish. DiIC_12_(3) was extracted from the same fish and analyzed by HPLC. The amount of food ingested was estimated from the quantities of DiIC_12_(3) extracted. The FI values correlated with the amount of food ingested (*r*^2^ = 0.78).

It is obvious that medaka fish utilize their visual and olfactory systems to search for food. They are usually more attracted to food floating on the surface of the water than that at the bottom, possibly because of the optical contrast between the food and the surface. This implied that it was necessary to design a food that possessed buoyancy in order to lure the fish. Fish also respond to chemical stimuli from a distance. For example, if a solution containing amino acids is dropped into the water, they respond by swimming vigorously and searching for food (Carr *et al.* 1977; Hara 2006; Lindsay & Vogt 2004). This results from the rapid diffusion and detected of the chemicals by the fish olfactory system. Because our aim was to evaluate the behavior triggered by taste stimuli, we required a food that retained its chemical compounds until it came into contact with the fish's taste buds. Our porous food matrix, consisting of starch, detergent and lipids (Fig. 2a, i), satisfied both these demands. The resulting food had a lower specific gravity than water, and its multiple cavities retained tastants and allowed them to be released in response to mechanical stimuli. In addition, the amphipathic matrix was labeled with a fluorescent dye, namely DiIC_12_(3), to allow quantification of the amount of food ingested (Fig. 2a, ii and iii).

Fish were fed the experimental food at 20 dpf and observed under a fluorescence microscope. The red fluorescence of DiIC_12_(3) could be detected in the abdominal region of fish that consumed the food (Fig. 2b, upper panels) but not in those that did not (Fig. 2b, lower panels). This experiment was performed in an entirely noninvasive manner, and therefore, the fluorescence was detected only in the gastrointestinal tract and clearly not on the body surface. Based on the fluorescence image of each fish, we were able to obtain a FI value by integrating pixel values. To confirm that this FI value represents the amount of food ingested by the fish, we compared the FI values with the amount of DiIC_12_(3) extracted from the body of the fish (*n* = 30). Fish were fed the foods containing tastants as described in the next section, and the FI value for each fish was measured. Subsequently, DiIC_12_(3) was extracted from the body of each fish by an organic solvent for quantification by HPLC analysis. As shown in Fig. 2c, a positive correlation was observed between the FI values and the amount of food ingested (*r*^2^ = 0.78).

### Effect of the tastants present in the fluorescently labeled food on fish behavior

Several varieties of tastants were added to the food to examine their effect on the feeding behavior of fish. Previous reports have shown that the taste system in fish is sensitive to amino acids and nucleotide-related substances (Kasumyan & Doving 2003; Kiyohara *et al.* 1975; Kohbara *et al.* 1992). We therefore used a mixture of glycine, l-serine, l-proline, l-glutamate sodium salt and IMP disodium salt as preferable tastants. In contrast, few studies describe the taste nerve responses to aversive tastants, such as quinine hydrochloride, caffeine and denatonium benzoate (Ogawa *et al.* 1997). With regard to behavioral responses, it is reported that pellets flavored with quinine hydrochloride are rejected by goldfish (*Carassius auratus*) (Lamb & Finger 1995) and we observed that zebrafish consumed less food containing denatonium benzoate than that containing no tastants (Oike *et al.* 2007). Because medaka fish have been seen to strongly avoid a solution of denatonium benzoate that is a potent bitter chemical for mammals, this compound was used as an aversive tastant (data not shown). The tastants were mixed with food materials (see *Materials and Methods*), and the resulting products were designated as amino acid–IMP (AN food) and denatonium foods (DN food). Food containing no tastants (NT food) was used as the control. With regard to the initial sequences of feeding behavior, no between-food differences were observed.

**Figure 3:**
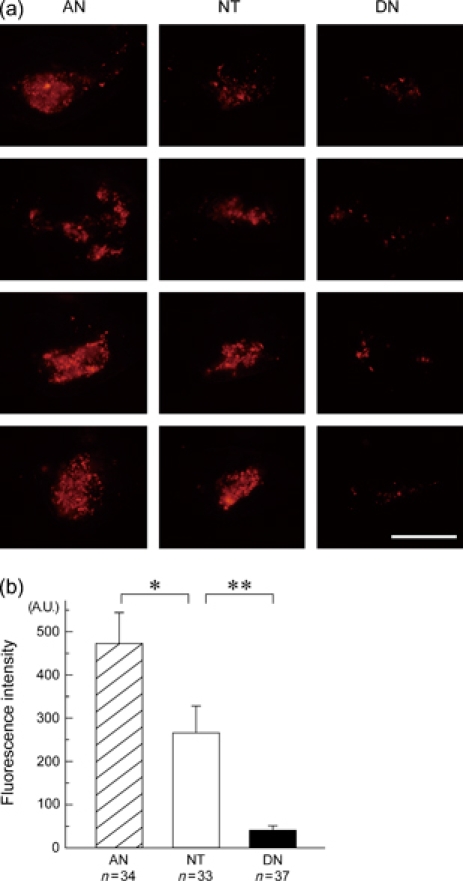
Effects of tastants on the feeding behavior of medaka fish (a) Fluorescence images of fish that were fed fluorescent dye-labeled foods containing different tastants. Only the abdominal regions are shown. Panels: AN column, fish that were fed AN food; NT column, those that were fed NT food; DN column, those that were fed DN food. The fish in the AN food group exhibited more intense fluorescence signals than those in the NT food group. The fish in the DN food group showed weak signals. Scale bar: 500 μm. (b) FI detected in fish that were fed AN, NT and DN foods. FI values were obtained from four sets of experimental groups, each of which contained 10 fish. The total number of fish examined is indicated. Bars represent the mean FI values with standard errors. Significant differences were observed between AN and NT (*P < 0.05) and between NT and DN (**P < 0.01).

Typical fluorescence images of fish after food ingestion are shown in Fig. 3a. Stronger intensities of fluorescence were detected in the fish that were fed AN food (panels in the AN column) than in those fed NT food (panels in the NT column). In contrast, only faint fluorescence images were observed for the group fed DN food (panels in the DN column). When the mean FI values were compared by Dunnett's multiple comparison test, significant differences were observed between the AN food group and the NT food group (*t* = 2.68, df = 101, *k* = 3, *P* = 0.016) and also between the NT food group and the DN food group (*t* = −2.99, df = 101, *k* = 3, *P* = 0.007). These data clearly indicate that medaka fish prefer AN food and are averse to DN food. Next, we prepared foods containing denatonium benzoate at lower concentrations by dipping NT food into a 10^−8^, 10^−6^ or 10^−4^m denatonium benzoate solution (see *Materials and Methods*). The foods were designated as 10^−8^ DN, 10^−6^ DN or 10^−4^ DN food and subjected to the behavioral assay together with NT^#^ food as a control, which was prepared by dipping NT food into water. The amount of ingested food decreased as the denatonium benzoate concentration increased, and a significant difference was detected between NT^#^ food and 10^−4^ DN food at *P* < 0.05 (Dunnett's multiple comparison test).

### Generation of transgenic fish expressing a dominant-negative mutant of Gα_i2_ in taste receptor cells

To examine whether the observed food preference behavior was dependent on the taste system of medaka fish, we used a transgenic approach to inhibit the taste receptor signal *in vivo*. In mammals and teleosts, two discrete subsets of taste-receptor-expressing cells are present; the T1R and T2R subsets. These two subsets of cells also express PLC-β2, a common taste signal effector enzyme (Adler *et al.* 2000; Ishimaru *et al.* 2005; Zhang *et al.* 2003). *In vitro* studies have shown that PLC-β2 can be activated by both G-protein α subunits of the alphaq family and β/γ subunits that have lower specificities (Hepler *et al.* 1993; Rhee 2001; Smrcka & Sternweis 1993). In mammals, however, taste receptor cells predominantly express Gα_gust_ and Gα_i2_ that lack the potency to activate PLC-β2 by themselves (Kusakabe *et al.* 2000; McLaughlin *et al.* 1992). Therefore, it is highly possible that PLC-β2 is activated by β/γ subunits released from Gα in taste receptor cells (Blake *et al.* 2001; Huang *et al.* 1999). We hypothesized that the inhibition of Gβ/γ-dependent activation of PLC-β2 in medaka taste receptor cells may cause a defect in taste signal transduction. To address this, we employed a rat Gα_i2_ dominant-negative mutant (Gα_i2_S47C) that was developed for the purpose of suppressing the GPCR signal to PLC-β2 by GTP-independent and irreversible binding to Gβ/γ (Slepak *et al.* 1995). The Gα_i2_S47C cDNA was fused to the medaka *plc-β2* promoter that can induce transgene expression specifically in taste receptor cells (Fig. 4a) (Aihara *et al.* 2007). The construct also contained the *gfp* gene, under the control of the same promoter, to facilitate the optical identification of transgenic fish. As a result, the F_3_ transgenic larvae exhibited GFP signals in the oropharyngeal region (Fig. 4b), exactly reproducing the pattern obtained with the transgene containing a single promoter and *gfp* reporter (Aihara *et al.* 2007). Next, we examined whether these transgenes were expressed in the same way as the endogenous *plc-β2* gene, by sequential observation of GFP fluorescence and the *in situ* hybridization signal (Fig. 4c). The upper panels of Fig. 4c show the colocalization of GFP and the endogenous *plc-β2* transcript, while the lower panels confirm the coexpression of the two transgenes, namely *gfp* and *gα_i2_S47C*. No signal was observed in wild-type fish sections hybridized with the rat *gα_i2_S47C* probe (data not shown), and therefore, cross-hybridization to endogenous medaka *gα* genes was negligible. The transgenic line established was named G_i2_S47C, and these fish were subjected to the feeding assay.

### Taste preference of G_i2_S47C transgenic fish

**Figure 5:**
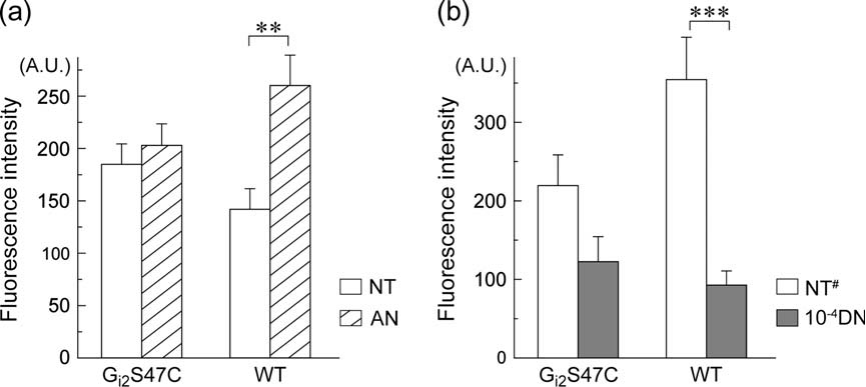
Differences in the feeding behavior between transgenic fish and wild-type fish (a) The feeding behavior for AN food. Bars represent the mean FI values with standard errors. Twenty-nine transgenic and 28 wild-type fish were used for the statistical analysis of NT and AN foods. A significant between-food difference was observed only in wild-type fish (**P < 0.01). (b) The feeding behavior with 10^−4^ DN food. For statistical analysis, 19 transgenic fish were used for both NT^#^ and 10^−4^ DN foods, and 18 and 19 wild-type fish were used for NT^#^ and 10^−4^ DN foods, respectively. A significant between-food difference was observed only in wild-type fish (***P < 0.001).

The taste preference of G_i2_S47C fish for AN food was examined (Fig. 5a), and the FI values for AN and NT foods were measured in transgenic and wild-type siblings. Two-way anova with genotype and food type as factors showed a significant interaction between factors [interaction *F*_1,113_ = 3.98, *P* < 0.05; genotype *F*_1,113_ = 0.08, *P* > 0.05; food *F*_1,113_ = 7.37, *P* < 0.01]. When the consumption for AN food and NT food was compared, a significant difference was detected in the case of wild-type fish (Aspin–Welch's *t*-test, *t* = −0.50, df = 50, *P* > 0.05) but not in transgenic fish (*t* = −3.37, df = 48, *P* < 0.01). G_i2_S47C and wild-type fish were examined for their aversive behavior toward 10^−4^ DN food (Fig. 5b). The FI values for 10^−4^ DN and NT^#^ food were measured in transgenic and wild-type siblings. As expected, the transgenic fish ingested a certain amount of 10^−4^ DN food. In addition, the use of two-way anova with genotype and food type as factors showed evidence of a significant interaction [*F*_1,74_ = 5.33, *P* < 0.05; genotype *F*_1,74_ = 2.17, *P* > 0.05; food *F*_1,74_ = 25.2, *P* < 0.001]. When the consumption for 10^−4^ DN food and NT^#^ food was compared, no significant difference was detected in the case of transgenic fish (Aspin–Welch's *t*-test, *t* = 1.97, df = 30, *P* > 0.05). In contrast, wild-type fish showed a highly statistically significant tendency to reject 10^−4^ DN food (*t* = 5.08, df = 22, *P* < 0.001). Taken together, it is concluded that G_i2_S47C transgenic fish had lost the ability to show food preferences and aversion because of the inhibition of inner-cellular taste signal transduction.

## Discussion

In this study, we have generated a transgenic medaka fish in which taste receptor-dependent PLC-β2 activation is inhibited. We have also developed an assay system that facilitates the quantification of the food preference behavior of this species. The transgenic fish exhibited a taste-blind phenotype, which showed neither a preference for food containing an amino acid–IMP mixture nor an aversion to that containing denatonium benzoate. This paper is the first to describe the genetic manipulation of the taste signaling pathway and to evaluate the behavioral effect of this manipulation in a fish model.

The feeding behavior of fish consists of multiple sequential steps: (1) noticing food, (2) approaching it, (3) capturing it and (4) ingesting/ejecting it. Both the olfactory and the taste systems are involved in steps 1, 2 and 3 (Bardach *et al.* 1967; Valentincic & Caprio 1994), while the taste system plays a major role in step 4 (Atema 1971). Previous studies that evaluated taste preferences in fish were performed by observing food sorting behavior or food consumption (Kasumyan & Morsy 1996; Lamb & Finger 1995), but these methods had experimental limitations with regard to the quantification of food intake. In the present study, we confronted this limitation by incorporating the fluorescent probe into the food, thereby allowing a direct measurement of the amount of food ingested. Moreover, the transparency of the juvenile fish allowed us to observe the food in the gastrointestinal tract and quantitatively assess its amount by fluorescence imaging (Fig. 2). In the sequence of feeding behavior, tastants stimulate olfactory as well as taste systems and indeed, compared with their taste nerves, fish olfactory nerves respond to amino acids at a lower threshold (Hara 1994). In this study, we intended to efficiently stimulate only the fish taste system. Because medaka fish seek food floating on the surface of the water, our food particles were designed to contain fine cavities for buoyancy. Moreover, these cavities, with the aid of tripalmitin, served to retain the tastants. The lipid also prevented the food from dissolving in the water. These physical characteristics allowed the tastants to stimulate the taste buds of the fish the moment it bit into the food. This design of the food matrix is also applicable for use with different tastants. For example, organic acids and salts are candidate materials for use in future experiments. The matrix is amphipathic, and therefore also allows the creation of foods containing fatty acids as tastants, whose receptor molecule still remains to be identified.

**Figure 6:**
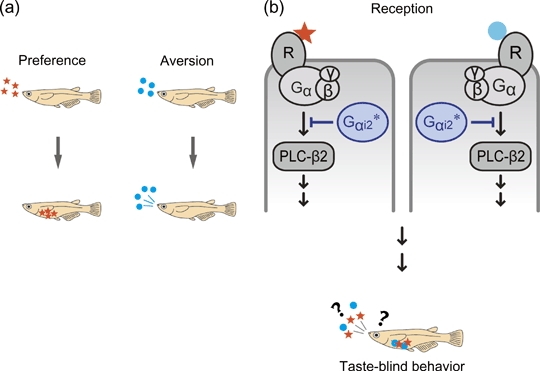
Transgenic medaka fish with suppressed PLC-β2 activation in taste receptor cells lose their ability to discriminate between taste modalities (a) Our behavioral assay system revealed that medaka fish prefer foods containing amino acids and IMP (red stars) and that they are averse to those containing denatonium benzoate (cyan circles), which are perceived as bitter tasting by mammals. (b) The transgenic medaka fish constructed in the present study was found to be taste blind. Because the Gα_i2_S47C mutant (Gα_i2_*) expressed in the taste receptor cells inhibits the signal transduction to PLC-β2, the fish were no longer able to recognize preferable or aversive tastes.

An amino acid–IMP mixture was used as a preferable tastant, and denatonium benzoate was used as an aversive tastant for medaka fish (Fig. 6a). These tastants also stimulate the mammalian taste system in which T1R and T2R function as taste receptors at the peripheral end of the system. T1R and T2R exhibit mutually exclusive expression patterns and different response profiles in the taste buds, and it is therefore likely that these taste receptors are responsible for discriminating between preferable and aversive tastes (Mueller *et al.* 2005). Despite the sharp contrast between the functions of these two receptors, they are both coexpressed with the same effector molecule, PLC-β2. It has previously been shown that mice lacking the *plc-β2* gene have neither a preference for amino acids nor an aversion to denatonium benzoate (Zhang *et al.* 2003). This result resembles the phenotype exhibited by G_i2_S47C transgenic fish in the current study (Fig. 5). We have recently shown that fish T1R is activated by all four amino acids used in this study, namely glycine, l-serine, l-proline and l-glutamate sodium salt, and that fish T2R is activated by denatonium benzoate (Oike *et al.* 2007). This result leads to the conclusion that the taste-blind phenotype of G_i2_S47C transgenic fish results from the inhibition of both the T1R and the T2R signaling pathways (Fig. 6b). Biochemical and electrophysiological data suggested the existence of ion channels in fish taste tissue, which are directly activated by l-proline (Kumazawa *et al.* 1998). However, based on the phenotype of our transgenic fish created by the inhibition of the GPCR-triggered pathway in taste bud cells, it is concluded that this type of channel is less responsible for the behavioral preferences shown toward amino acids. Taken together, it is likely that teleosts and mammals share a common molecular strategy for discriminating between the two taste modalities mediated by T1R and T2R.

It is noteworthy that our behavioral assay system was able to detect a phenotype that potentially resulted from the compromised function of a single gene product, PLC-β2. Preference–aversion discrimination may indeed be difficult to study because it involves a series of integrated processes. These processes include signal input at the taste buds, signal transmission from the taste nerves to the central nervous system for cognition and signal output for feeding behavior. When used in combination with forward genetics or gene manipulation, our assay system provides a method for dissecting these processes at the molecular level. The analysis of taste systems in small fish as model animals should contribute to our understanding of the taste transduction mechanisms in vertebrates, including humans.
